# ROS-Induced Autophagy of Skeletal Muscle Confers Resistance of Rice Flower Carp (*Cyprinus carpio*) to Short-Term Fasting

**DOI:** 10.3390/genes15070840

**Published:** 2024-06-26

**Authors:** Jia Cheng, Junhan Luo, Ziyang Xu, Zhouying Liu, Lingsheng Bao, Liangyi Xue

**Affiliations:** 1 School of Marine Sciences, Ningbo University, Ningbo 315832, China; 2 College of Biological and Chemical Engineering, Hunan Engineering Technology Research Center for Amphibian and Reptile Resource Protection and Product Processing, Changsha University, Changsha 410022, China

**Keywords:** short-term fasting, autophagy, skeletal muscle, rice flower carp, oxidative stress

## Abstract

Starvation is one of the main stresses for fish due to food shortage, the evasion of predators, and intraspecific competition. This research evaluated the impact of brief fasting periods on reactive oxygen species (ROS) levels, antioxidant response, mRNA expression of antioxidants, autophagy-related signaling genes, and autophagosome development in the muscle tissue of rice flower carp. Following a three-day fasting period, the levels of ROS and MDA rose. Additionally, after 3 d of fasting, there was a notable upregulation of *NRF2* and significant increases in the levels of GSH and the activities of enzymes such as SOD, CAT, GST, GR, and GPX, while the expression of the autophagy marker gene *LC3B* did not change (*p* < 0.05). After 7 d of fasting, the content of the ROS, the activity of SOD and GR, and the GSH content reached the maximum (*p* < 0.05). Concurrently, there was a significant rise in the quantity of autophagosomes. An RT-qPCR analysis revealed that seven d of starvation significantly elevated the mRNA expression of genes associated with the initiation and expansion of autophagosome membranes, vesicle recycling, and cargo recruitment, including *ULK1*, *BECLIN1*, *LC3B*, *ATG3*, *ATG4B*, *ATG4C*, *ATG5*, *ATG9*, and *P62*. After feeding resumed for 3 d, the mRNA level of *BECLIN1*, *ATG3*, *ATG4B*, *ATG4C*, *ATG5*, *LC3B*, and *P62* still remained at a high level. The LC3II protein reached its highest level. All autophagy-related gene expression decreased in the 7-day resumed feeding group. Our data implied that short-term fasting can cause oxidative stress and disrupt the antioxidant system first and then induce autophagy in the muscles of rice flower carp. These findings shed light on how fasting affects muscle homeostasis in fish. ROS-induced autophagy of the skeletal muscle may confer the resistance of rice flower carp to short-term fasting.

## 1. Introduction

In nature, starvation is one of the main stresses for fish due to food shortage, the evasion of predators, and intraspecific competition caused by seasonal changes [1,2,3]. During the initial phase of starvation stress, fish undergo various changes, including alterations in enzyme activity and hormone levels, to modify their metabolic rate and cope with the lack of food [4]. As the duration of starvation extends, fish are unable to sustain bodily homeostasis, leading to abnormalities in several physiological and biochemical markers [5]. The research has indicated that an overabundance of reactive oxygen species (ROS) can directly trigger oxidative stress [6]. A moderate amount of ROS helps regulate cell survival signaling, but too many ROS can trigger oxidative stress, causing DNA hydroxylation, tissue damage, and protein denaturation [7].

Active oxygen ROS also undergo a peroxidation reaction with lipids to produce lipid peroxidation products that are harmful to the body, such as malondialdehyde (MDA); MDA is a marker of oxidative lipid damage in the body.

When ROS levels are too high or their removal is compromised, the body’s defense mechanism will initiate an antioxidant response to eliminate the surplus ROS and prevent further damage. Fish have a complex antioxidant defense system, which contains a variety of antioxidant enzymes (SOD, CAT, POX, GST, GPX, GR, etc.) and non-enzyme small molecules [8]. Morales et al. observed that antioxidative enzyme activity, including SOD, CAT, and GPX, markedly increased in fish undergoing starvation. The study also showed that oxidative damage was reversible, as all biomarkers measured returned to baseline levels after the fish were refed [9].

An overaccumulation of ROS may activate pathways associated with *NRF2*, engaging elements like *FOXO3a* and *Keap1*. These elements either control oxidative/antioxidant dynamics or impact cellular redox states directly [10]. The *NRF2* is a transcription factor of nuclear factor erythroid 2 (*NF-E2*). It is a key element in the endogenous antioxidant system. It regulates the antioxidant system under oxidative stress. Typically, NRF2 connects with the antioxidant response element (ARE), and the NRF2/ARE pathway is crucial for the body’s antioxidant defense mechanism. In addition, *NRF2* works in combination with its negative regulatory protein *KEAP1* in the cytoplasm. Jiang W D found that Cu^2+^ can induce oxidative stress in fish, interfere with the function of the antioxidant system, and change the *NRF2-ARE* antioxidant signaling pathway. However, there is limited research on how starvation impacts the adaptive responses in fish muscle. The involvement of the KEAP1-NRF2-ARE pathway in oxidative stress due to fasting is not well understood and requires further investigation, particularly in aquatic organisms.

Antioxidant systems and autophagy are crucial for fish in resisting environmental stress. ROS and autophagy play a part in complex signal transduction processes and various interactions [11,12]. Autophagy is a cellular mechanism where eukaryotic cells employ lysosomes to break down organelles and proteins, thereby sustaining metabolic equilibrium [13,14]. The activation of autophagy is strictly regulated, such as the mammalian rapamycin target protein (mTOR) signal pathway, the phosphatidylinositol 3-kinase (PI3K) pathway, the adenylate-activated protein kinase (AMPK) signal pathway, etc. [15]. At present, there are more than 40 autophagy-related genes that have been identified, which can be grouped into five categories based on their functions: the Atg1 subfamily, Atg9 subfamily, phosphatidylinositol-kinase subfamily, Atg12-Atg5 subfamily, and Atg8 subfamily [16]. 

Autophagy is crucial when there is a shortage of energy. Takeshi et al. starved the cultured zebrafish cells and found that the lack of amino acids promotes the breakdown of long-lived proteins. It also found that the autophagy process is regulated by the P13K class III pathway [17]. The study of rainbow trout muscle also indicates that autophagy was induced after fasting for 14 d. Autophagy has an impact on ROS levels, potentially breaking them down to prevent cellular damage from toxicity [18]. On the other hand, studies suggest that autophagy activation can lead to higher ROS levels, showing a robust link between autophagy and ROS [19]. This relationship involves complex mechanisms beyond straightforward activation or suppression, suggesting that more investigation is needed. 

In vertebrates, skeletal muscle is the primary organ for motor responses and plays a crucial role in both material and energy metabolism [20]. The impact of starvation on the autophagic pathway in fish muscle is not well understood and requires further study. Rice flower carp (*Cyprinus carpio*) is a characteristic fish in Quanzhou City, Guangxi Province, and is also a typical species under the rice and fish integrated cultivation model, which has been widely popularized.

Therefore, in this study, we focus on how fish muscle adapts to short-term fasting through oxidative stress and autophagy. This research also explores multi-level regulation and signal transduction in fish muscle to understand how oxidative stress affects rice flower carp, aiming to mitigate oxidative damage.

## 2. Methods and Materials

### 2.1. Fish Sampling and Tissue Preparation

The fish for this study were sourced from a fish farm. A total of 45 healthy specimens, averaging 120.34 ± 36.72 g, were randomly allocated into five groups, each placed in separate ponds. These groups experienced fasting for 0, 3, or 7 d. After fasting, they resumed regular feeding for 3 or 7 d. Samples from the fast muscle of 9 live fish were collected and kept in a freezer at −80 °C. All the procedures were conducted under anesthesia with sodium pentobarbital to ensure minimal distress to the fish.

### 2.2. RNA Extraction and Complementary DNA Synthesis

The muscle tissue mRNA was isolated with the Trizol reagent kit (Takara Bio Co., Dalian, China) adhering to the manufacturer’s guidelines. The purity and concentration of the RNA were determined by analyzing the 260/280 nm absorbance ratio using a Nanodrop 2000 spectrometer (Nanodrop Technologies, Wilmington, DE, USA). Then, 1.5% agarose gel electrophoresis was used to verify the integrity and relative amount of the RNA. Complementary DNA (cDNA) was created with the PrimeScript TM reagent kit and the Eraser gDNA kit (Takara Bio Co., Dalian, China).

### 2.3. Quantitative Real-Time PCR (qPCR)

The primer pairs can be found in Table 1. *Rpl13* served as an internal reference. Quantitative real-time PCR (qPCR) was performed using the Bio-Rad CFX96TM system from the USA. The reaction mixture totaled 25 µL, composed of 12.5 µL SYBR Premix Ex Taq (Takara Bio Co., Dalian, China), 10.5 µL RNase-free water, 1 µL cDNA templates, and 0.5 µL of both forward and reverse primers. Amplification proceeded at 95 °C for 30 s, followed by 40 cycles at 95 °C for 5 s and 58 °C for 25 s. The melting curve analysis ranged from 65 °C to 95 °C, increasing in increments of 0.5 °C to measure the fluorescence sequentially.

### 2.4. ROS, MDA, and Enzyme Activity

The content of the ROS and malondialdehyde (MDA) were determined by using detection kits (Nanjing Jiancheng Bioengineering Institute, Nanjing, China) following the product instructions. The activity of all the enzymes was also identified by enzyme-detection kits (Nanjing Jiancheng Bioengineering Institute, Nanjing, China). The T-SOD activity was detected under 550 nm and the GPX and GST activities at 412 nm. The UV detection wavelength of CAT and GSH was 405 nm.

### 2.5. Transmission Electron Microscopy (TEM)

The muscle tissue samples were rinsed and then sliced into 2 mm × 2 mm sections and then fixed by fixed solution. An H7600 electron microscope (Hitachi Ltd., Tokyo, Japan) was employed to observe the formation of autophagosomes.

### 2.6. Western Blot Analysis

A 0.1 g sample of tissue was combined with 1 mL of lysate (containing 50 mM Tris HCl, 150 mM NaCl, 1 mM EDTA, 1 mM PMSF, 1% NP-40, and 0.5% sodium deoxycholate) to fully lyse the proteins. Following homogenization, the homogenates were centrifuged at 12,000× *g* for 10 min at 4 °C. The total protein content in the supernatant was measured using the Bio-Rad protein assay kit (Bio-Rad, Hercules, CA, USA). The protein samples (40 μg per lane) were resolved through SDS-PAGE and then moved onto a 0.45 μm PVDF membrane for the Western blot analysis. The membrane was blocked for one hour and then incubated with the primary antibody overnight at 4 °C. The anti-LC3A/B antibody (ab128025) was used at a 1/1000 dilution for probing, while anti-β-actin (ab8224) at a 1/1000 dilution functioned as a loading control. The protein levels were measured using densitometric scanning and then adjusted relative to the loading control.

### 2.7. Statistical Analysis

The data were shown as the means ± SEM. The significance of the results was determined using a one-way analysis of variance (ANOVA), followed by Tukey’s post hoc test. This analysis was performed with SPSS v16.0 (SPSS Inc., Chicago, IL, USA). A *p* value was employed to evaluate the significant difference between the groups (a *p* value of 0.05 or less represents the significant differences).

## 3. Results

### 3.1. ROS Content of Muscle after Fasting

The effect of fasting on the ROS content in the rice flower carp muscle is shown in Table 2. Following 3 d of fasting, the muscle ROS content was notably higher compared to the fish with normal feeding habits (*p* < 0.05). Upon 7 d of fasting, the ROS content increased to the highest level and gradually decreased after resuming feeding (*p* < 0.05).

### 3.2. mRNA Expression of Genes Linked to Antioxidant-Related Signaling in Rice Flower Carp Muscles after Fasting

**Figure 1:**
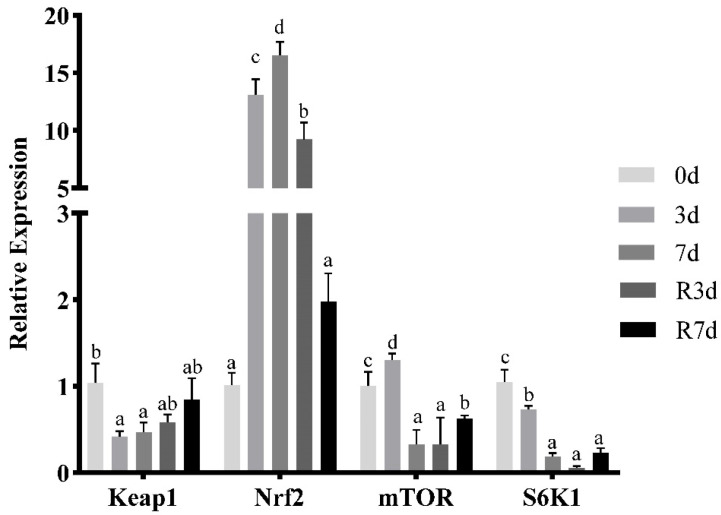
The expression of the antioxidant-related signaling molecules in the rice flower carp muscle during short-term fasting and resuming feeding. The values are shown as the mean ± SEM of the normalized transcript levels of each antioxidant-related signaling molecule (*n* = 3 replicate tanks), 3 fish were sampled for each tank, and the data with different letters indicate a significant difference (*p* < 0.05).

In the muscle, as shown in Figure 1, the expression of *NRF2* was upregulated (*p* < 0.05) after 3 d of fasting, reached the peak after fasting for 7 d, and decreased gradually after 3 and 7 d of refeeding. mTOR expression showed a significant increase after 3 d of fasting (*p* < 0.05), then decreased apparently following 7 d of fasting and 3 d of refeeding (*p* < 0.05), and recovered after refeeding for 7 d (*p* < 0.05). The expression level of *Keap1* and *S6K1* decreased significantly (*p* < 0.05) during fasting and increased after refeeding for 7 d, but the increase was not significant.

### 3.3. Antioxidant Parameters after Fasting in Rice Flower Carp Muscles

The effect of fasting on the antioxidant parameters in the muscle is shown in Table 2. After 3 d of fasting, the MDA content in the muscle was considerably higher than that in the normally fed group. The peak was reached after 7 d of fasting (*p* < 0.05) and then decreased gradually in the refeeding groups (*p* < 0.05). After fasting for 3 d, the activity of SOD, CAT, GST, GPX, and GR and the content of GSH were significantly upregulated (*p* < 0.05). Among them, the activity of CAT, GST, and GPX reached the peak at 3 d for the fasting group (*p* < 0.05), while the activity of SOD and GR and the GSH content reached the maximum after 7 d of fasting (*p* < 0.05). After resuming feeding, their activity reduced by varying degrees. The activities of these enzymes increased significantly after resuming feeding. The activity of SOD, CAT, GST, and GR and the GSH content decreased significantly at 3 d for the refeeding group (*p* < 0.05), while the GPX activity remained the same at 7 d for the fasting group. After 7 d of resuming feeding, all the antioxidant parameters further decreased, and the content of MDA and GSH and the GST activity returned to normal levels. The activity of SOD, CAT, GPX, and GR was higher than the normally fed group.

**Figure 2:**
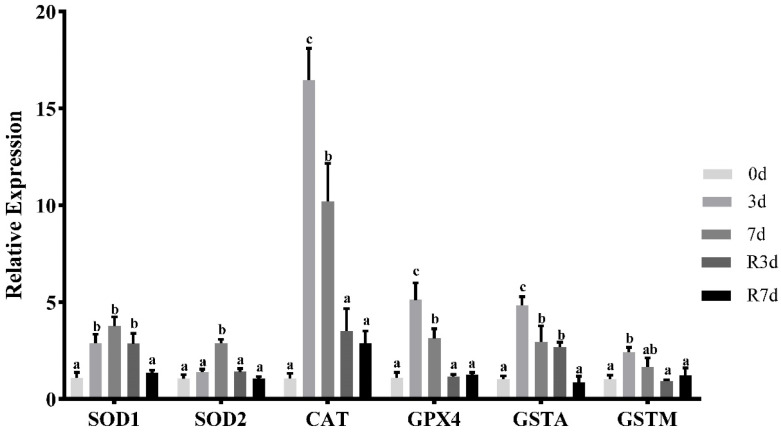
The expression of the antioxidant enzymes in rice flower carp. The muscle during short-term fasting and resuming feeding. The values are shown as the mean ± SEM of the normalized transcript levels of each antioxidant enzyme (*n* = 3 replicate tanks), 3 fish were sampled for each tank, and the data with different letters indicate a significant difference (*p* < 0.05).

Figure 2 illustrates how fasting impacted the expression of the antioxidant enzymes in rice flower carp. After 3 d of short-term fasting, the expression levels of the *SOD1*, *CAT*, *GPX4*, *GSTA*, and *GSTM* genes increased significantly (*p* < 0.05). Upon 7 d of fasting, the mRNA level of *SOD1* and *GSTM* remained at high levels and the expression of *SOD2* rose significantly, while the mRNA levels of *CAT*, *GPX4,* and *GSTA* were significantly lower than that of the 3-day fasting group (*p* < 0.05). Three days after resuming feeding, the expression of SOD2, CAT, GPX4, and GSTM tended to decline toward normal feeding levels, while the mRNA levels for SOD1 and GSTA remained elevated (*p* < 0.05) but decreased after 7 d of resuming feeding (*p* < 0.05).

### 3.4. Effects of Fasting on Autophagosome Formation in Rice Flower Carp Muscle

**Figure 3:**
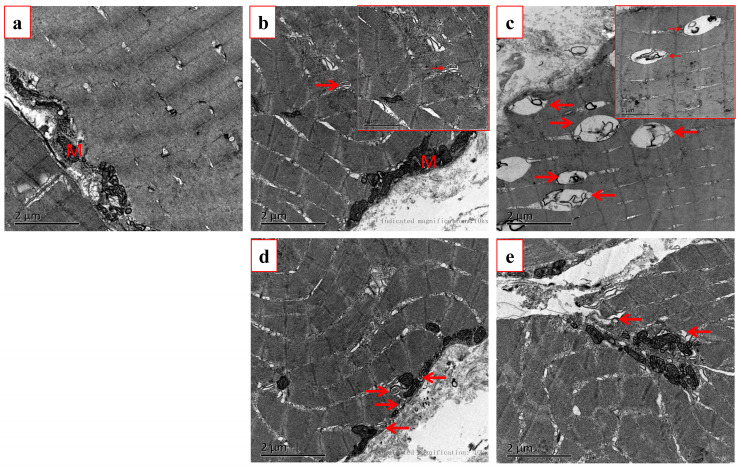
An autophagosome identified by using electron microscopy in the muscle of rice flower carp. (**a**) Starvation for 0 d, (**b**) fasting for 3 d, (**c**) fasting for 7 d, (**d**) recovery feeding for 3 d, and (**e**) recovery feeding for 7 d. The arrow points to the autophagosomes, M indicates mitochondria.

The impact on the autophagosome formation in the muscle tissue was examined using a transmission electron microscope (Figure 3). Figure 3a displays the muscle from the normally fed fish, with minimal evidence of autophagosomes. After a 3-day fast, autophagosomes began to appear in the muscle tissue (Figure 3b), with an increase in their number. Additionally, after 7 d of fasting, more autophagosomes were visible (shown by the arrows in Figure 3c). Additionally, a lot of autophagosomes were observed after the fish had resumed feeding for 3 d (Figure 3d). And several autophagosomes can be found at 7 d for the fasting group (Figure 3e).

### 3.5. mRNA Expression of Autophagy-Related Genes

**Figure 4:**
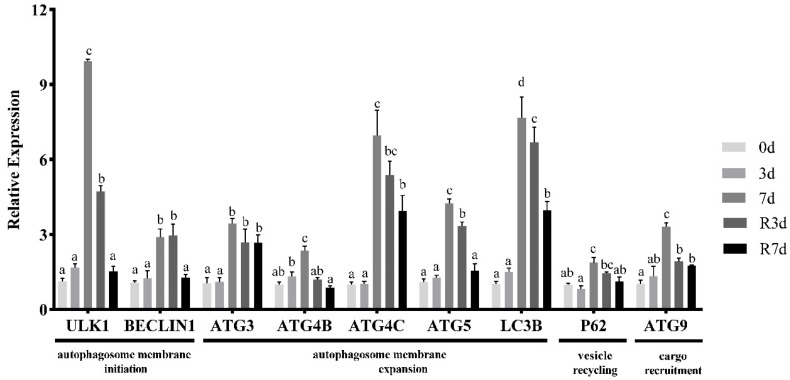
The expression of the autophagy and autophagy-related genes in the rice flower carp muscle during short-term fasting and resuming feeding. The values are shown as the mean ± SEM of the normalized transcript levels of each autophagy-related gene (*n* = 3 replicate tanks), 3 fish were sampled for each tank, and the data with different letters indicate a significant difference (*p* < 0.05).

Figure 4 depicts the impact of fasting on the autophagy-related genes in the muscle of rice flower carp. An analysis of autophagy-related gene expression in the rice flower carp muscle revealed that most of the tested genes were not upregulated after a 3 d period of starvation. However, after a 7 d starvation period, the expression of the autophagosome membrane initiation genes (*BECLIN1* and *ULK1*), membrane expansion genes (*LC3B*, *ATG4B*, *ATG4C*, and *ATG5*), vesicle recycling gene (*P62*), and cargo recruitment gene (*ATG9*) in the muscle was significantly increased (*p* < 0.05). After feeding resumed for 3 d, the mRNA level of *BECLIN1*, *ATG3*, *ATG4B*, *ATG4C*, *ATG5*, *LC3B*, and *P62* still remained at a high level (*p* < 0.05). All autophagy-related gene expression decreased in the 7-day resumed feeding group (*p* < 0.05).

### 3.6. Conversion of LC3II during Fasting and Refeeding

**Figure 5:**
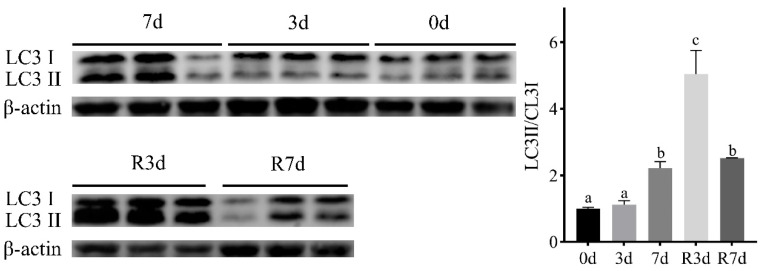
A Western blot analysis of the LC3 protein in the muscle of rice flower carp during short-term fasting and resuming feeding. In the figure above, from right to left, they were fasting for 0 d, fasting for 3 d, and fasting for 7 d. In the figure below, from left to right, they resumed feeding for 3 d and resumed feeding for 7 d. β-actin was used as the loading control. The values represent the mean ± SEM (*n* = 3 replicate tanks), 3 fish were sampled for each tank, and the data with different letters indicate a significant difference (*p* < 0.05).

The ratio of the LC3II to LC3I protein content is a crucial metric for assessing autophagy levels. This ratio significantly rises when autophagy is active. To determine if short-term fasting triggers autophagy, Western blotting was employed to examine the conversion of LC3II. As shown in Figure 5, the LC3II/LC3I ratio did not change significantly after 3 d of fasting (*p* < 0.05) but increased significantly after fasting for 7 d (*p* < 0.05). Moreover, the ratio reached the maximum after 3 d of resumed feeding and decreased after 7 d of resumed feeding.

## 4. Discussion

In this study, we assayed the possible mechanisms by which ROS mediates autophagy in rice flower carp muscle during short-term fasting. After short-term fasting for 3 d there was a mass production of ROS. In order to eliminate the effect of ROS, the antioxidant enzyme system and non-enzymatic antioxidant system were initiated, keeping the muscle in a redox-balanced state. Thus, we examined the expression of *KEAP1-NRF2*, the upstream regulator of the antioxidant-related signaling molecules. The mRNA expression of *NRF2* increased notably following 3 d of fasting, whereas the *KEAP1* levels dropped. The expression of the autophagy-related genes, however, stayed relatively stable. The results of the Western blot also confirmed this conclusion. Therefore, short-term fasting for 3 d has no significant effect on the autophagy of the muscle in rice flower carp. By using a transmission electron microscope, we can observe that the number of autophagosomes was increased but not significant. This may be because of the high efficiency of the antioxidant system in ROS elimination.

Following 7 d of fasting, NRF2 reached its peak expression, while Keap1 dropped to its lowest level. The SOD and GR activities, along with the GSH content, peaked, while the CAT, GST, and GPX activities reached their maximum after just 3 d of fasting. However, the ROS content kept rising. These results revealed that, upon 7 d of fasting, the oxidative scavenging ability of the muscle was exceeded, and the cells were damaged by oxidative stress [21]. Furthermore, a large amount of ROS was accumulated in the muscle and caused cell damage and then induced autophagy. In CHO (Chinese hamster ovary) cells, fasting led to increased ROS and H_2_O_2_ levels, which subsequently triggered autophagy. On the other hand, cysteine protease hsatg4 can eliminate the formation of autophagosomes and prevent protein degradation [22]. After a 7-day fasting period, there was a significant increase in the quantity of autophagosomes. With the fasting prolonged, the antioxidative system cannot relieve the ROS-mediated oxidative stress. As a result, the number of autophagosomes increases, and proteins begin to degrade. This trend was consistent with the results in rainbow trout muscle [22]. According to Rahman et al., the buildup of endogenous ROS triggered autophagy in C2C12 cells, a reaction to sudden nutrient scarcity and mTORC1 inhibition [11]. Additionally, research with HeLa cells indicates that overexpressing SOD can substantially decrease autophagy rates [23]. *ATG4* was identified as a direct target for ROS [24]. Studies of *Ctenopharyngodon idellus* by Wang et al. showed that O^2−^ is one of the major regulators of autophagy [24,25]. Also, we found that the expression of autophagy-related genes increased significantly following 7 d of starvation (*p* < 0.05). These genes are crucial for various stages of autophagy, encompassing the initiation, expansion, and formation of autophagosome membranes, as well as vesicle recycling [26]. The *ULK1* is crucial for the early stages of autophagosome formation, while *BECLIN1* is essential for initiating autophagy by generating the isolation membrane, a structure that encapsulates cytoplasmic components to form the autophagosome [27]. Our findings indicate that nutrient deprivation significantly affects the autophagic process in fish muscle. Likewise, Fan et al. (2020) discovered that fasting triggers the complete autophagic process [28]. In muscle tissue, activated autophagy breaks down cellular components, providing energy or substrates that support cell survival. *P62* acts as an important connector between autophagy and oxidative stress by interacting with *NRF2* [29]. Our findings show that P62 mRNA expression rose after 7 d of starvation in the muscle of rice flower carp, indicating that autophagy triggered by food deprivation could be associated with oxidative stress.

After resuming feeding for 3 d, the ROS level decreased significantly, indicating that the resumption of feeding can reduce the production of ROS. Meanwhile, the mRNA level of *BECLIN1*, *ATG3*, *ATG4B*, *ATG4C*, *ATG5*, *LC3B*, and *P62* still remained at a high level (*p* < 0.05). All autophagy-related gene expression decreased in the 7-day resumed feeding group (*p* < 0.05). The results observed were consistent with zebrafish after fasting reported by Huang et al [30]. We propose that ROS contribute to oxidative stress and significantly influence the mediation of autophagy during nutrient deprivation.

In summary, we propose that, under short-term fasting for 3 d, the ROS in rice flower carp muscle was accumulated and then the antioxidative systems were activated to eliminate the free radicals. With the fasting prolonged, the accumulation of ROS in muscles exceeded a certain level, the oxidative metabolism system experienced disruption, and it could not release high concentrations of O^2−^ and H_2_O_2_. It eventually led to oxidative damage and triggered widespread autophagy in the muscle of rice flower carp.

## Figures and Tables

**Table 1:** The primer sequences used in this study.

Gene	Primer (5′-3′)	Product (bp)
*ATG3* F	CGCCAGTTTTGAAGGAATCT	142
*ATG3* R	AGATAGGGCTTCACCTTTGCT	
*ATG4B* F	TAGGTGGGAAGCCGAACA	202
*ATG4B R*	CAGAAGAAGCCAGCAGCAA	
*ATG4C* F	CCGAGGCATTAGATGACGATA	119
*ATG4C* R	GATGGTGGAGCCAGGAAGA	
*ATG5* F	CGTTGGGAGACCTGCTGAA	180
*ATG5* R	TTGGTGCTGGGATGATGCT	
*CAT* F	AGCAGACACCGTCCGAGAT	200
*CAT* R	GCAAACTCCAGAAGTCCCAA	
*GPX4* F	AACCAGTTTGGAAAGCAGGAG	227
*GPX4* R	TTCACCACCTGACCTTCTCG	
*GSTA* F	AGACGACCCGACTGAACAAG	188
*GSTA* R	TCTCCAAGTATCCATCCCACA	
*GSTM* F	CTTGCTCAACCAATCCGTCT	188
*GSTM* R	GCATTGCTTTGGACCACTTT	
*ULK1* F	AGGGAGGAAGCCGTAGAAACA	161
*ULK1* R	ACAGTGGACAAAAGCCAAGGT	
*BECLIN1* F	AGGAGGTGAAGAGCGATAAGG	123
*BECLIN1* R	CCAGGCGACGGTTGTGA	
*LC3B* F	ACATTTGAGCAGCGGGTG	208
*LC3B* R	GGAAGAAAGCCTGATTGGAGT	
*SOD1* F	ATGGTGGACCGACGGATAGT	187
*SOD1 R*	TCTTCATTGCCTCCTTTACCC	
*SOD2* F	ACTACAGGTCTCGTCCCACT	123
*SOD2* R	GACATTCTCCCAGTTCACAA	
*Nrf2* F	TGCCCAATGAGAATCCCTT	166
*Nrf2* R	ATGGGACTTTACTACGGTGG	
*Keap1* F	TTTGCGGAGGAGATTGGC	202
*Keap1* R	TGACCCAAGCGACCCTAC	
*mTOR* F	AGGATGGCACCTGACTATGA	252
*mTOR* R	ATTTGAGCCCTGAGATGAAG	
*S6K1* F	CTGCTCCACATTAGACACCT	233
*S6K1* R	TCTTCCTGGGCTTTACATAC	
*P62* F	CTCTTTACCCTCACCTGCCTCA	101
*P62* R	AGTCAACCAGCCGCCTTCAT	
*ATG9* F	TCAATGAGTTGGACCACGAGC	151
*ATG9* R	GCGATGAGGACAGCCAGAAGA	
*RpL13* F	CAGAGGCTGAAGGAGTACCG	122
*RpL13* R	ATGACGGGTCCAGTAAGCTG	

**Table 2:** The antioxidant index in the skeletal muscle of rice flower carp.

Name	0 Day	3 Day	7 Day	R3	R7
ROS	100.03 ± 0.67 ^a^	119.42 ± 5.11 ^bc^	130.56 ± 4.29 ^c^	106.93 ± 3.52 ^ab^	107.98 ± 3.95 ^ab^
MDA	1.63 ± 0.28 ^b^	2.51 ± 0.20 ^c^	3.44 ± 0.14 ^d^	1.56 ± 0.29 ^b^	0.96 ± 0.086 ^a^
SOD	5.97 ± 0.45 ^a^	16.37 ± 0.34 ^c^	24.05 ± 2.08 ^d^	18.66 ± 0.97 ^c^	11.41 ± 0.73 ^b^
CAT	1.65 ± 0.54 ^a^	3.56 ± 0.96 ^e^	3.03 ± 0.07 ^d^	2.45 ± 0.13 ^c^	2.14 ± 0.10 ^b^
GST	10.91 ± 0.56 ^ab^	37.25 ± 8.51 ^d^	27.63 ± 1.93 ^cd^	17.28 ± 2.01 ^bc^	3.82 ± 0.85 ^a^
GPX	36.09 ± 1.55 ^a^	76.96 ± 3.22 ^d^	64.25 ± 2.11 ^c^	61.27 ± 2.15 ^bc^	54.20 ± 4.43 ^b^
GR	23.40 ± 2.23 ^a^	74.87 ± 2.74 ^c^	94.31 ± 6.04 ^d^	73.79 ± 7.82 ^c^	49.00 ± 4.55 ^b^
GSH	10.39 ± 0.15 ^ab^	25.17 ± 2.00 ^c^	52.55 ± 0.47 ^d^	13.73 ± 1.33 ^b^	7.55 ± 1.43 ^a^

The values represent the mean ± SEM (*n* = 3 replicate tanks); 3 fish were sampled for each tank. The values within the same row having different superscripts are significantly different (*p* < 0.05). ROS (% DCF florescen), MDA (nmol/mg prot), GST (U/mg prot), CAT (U/mg prot), SOD (U/mg prot), GSH (µmol/L prot), and GPX (U/mg prot).

## Data Availability

The original contributions presented in the study are included in the article, further inquiries can be directed to the corresponding author.
